# RETRACTION: Effects of Evidence‐Based Nursing Care Interventions on Wound Pain and Wound Complications Following Surgery for Finger Tendon Injury

**DOI:** 10.1111/iwj.70623

**Published:** 2025-04-21

## Abstract

**RETRACTION:** ZhangX.‐L. , WangC.‐Y. , PanL.‐L. , and LiY.‐J. , “Effects of Evidence‐Based Nursing Care Interventions on Wound Pain and Wound Complications Following Surgery for Finger Tendon Injury,” International Wound Journal 21, no. 3 (2024): e14818, 10.1111/iwj.14818.38444052 PMC10915127

The above article, published online on 05 March 2024, in Wiley Online Library (http://onlinelibrary.wiley.com/), has been retracted by agreement between the journal Editor in Chief, Professor Keith Harding; and John Wiley & Sons Ltd. Following an investigation by the publisher, all parties have concluded that this article was accepted solely on the basis of a compromised peer review process. The editors have therefore decided to retract the article. The authors did not respond to our notice regarding the retraction.

**RETRACTION:** X.‐L. Zhang , C.‐Y. Wang , L.‐L. Pan , and Y.‐J. Li , “Effects of Evidence‐Based Nursing Care Interventions on Wound Pain and Wound Complications Following Surgery for Finger Tendon Injury,” International Wound Journal 21, no. 3 (2024): e14818, 10.1111/iwj.14818.38444052 PMC10915127

The above article, published online on 05 March 2024, in Wiley Online Library (http://onlinelibrary.wiley.com/), has been retracted by agreement between the journal Editor in Chief, Professor Keith Harding; and John Wiley & Sons Ltd. Following an investigation by the publisher, all parties have concluded that this article was accepted solely on the basis of a compromised peer review process. The editors have therefore decided to retract the article. The authors did not respond to our notice regarding the retraction.

